# Supplementary figures and images for: HDAC inhibition delays photoreceptor loss in Pde6b mutant mice of retinitis pigmentosa: insights from scRNA-seq and CUT&Tag
Source: PeerJ. 2023 Jul 12;11:e15659. doi: 10.7717/peerj.15659 (PMC10349563; doi:10.7717/peerj.15659)

A

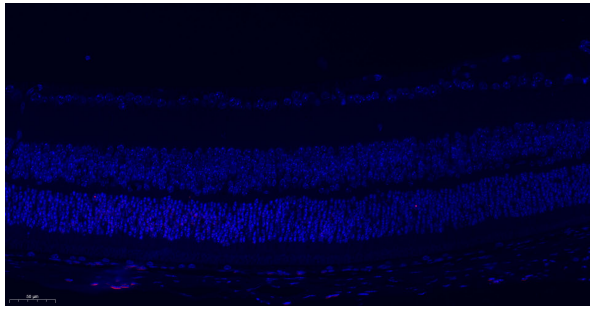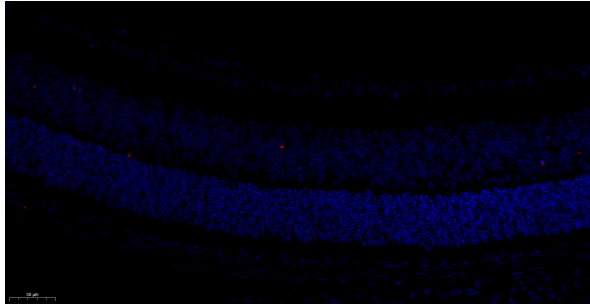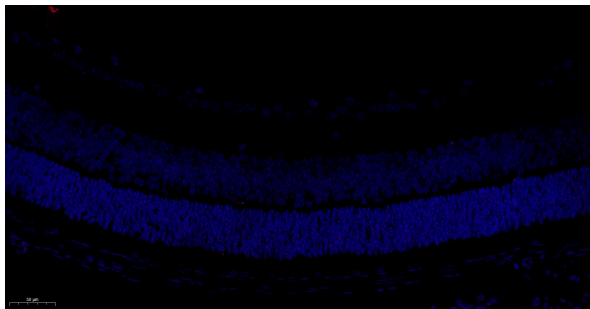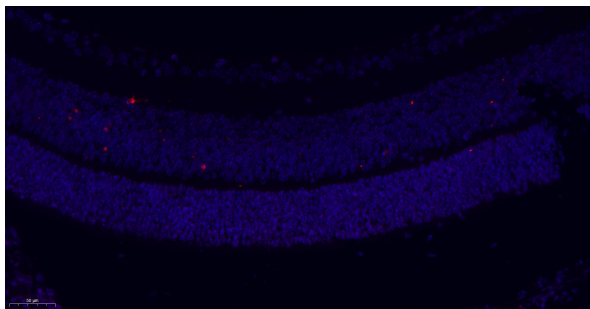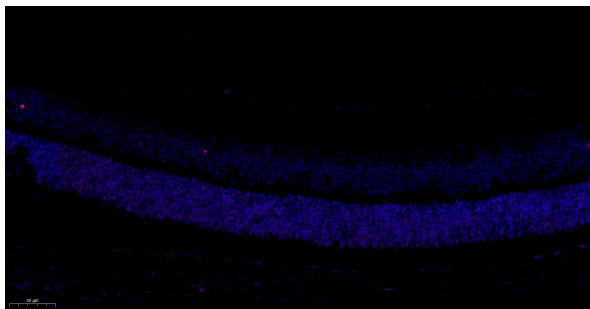

B

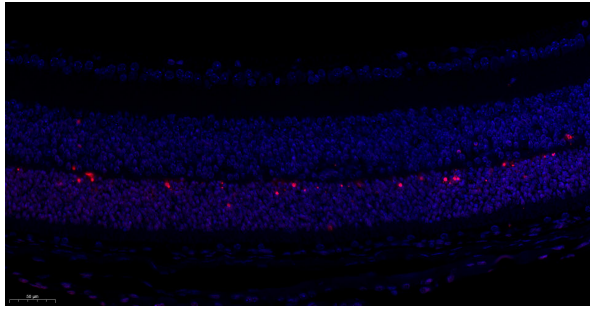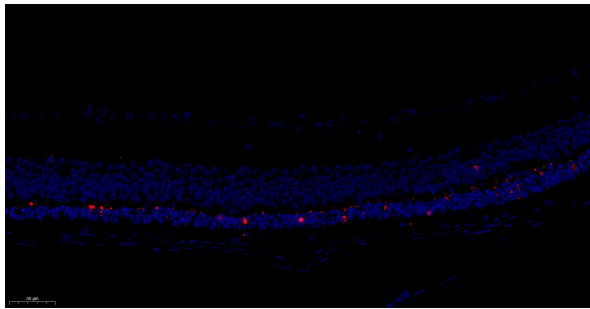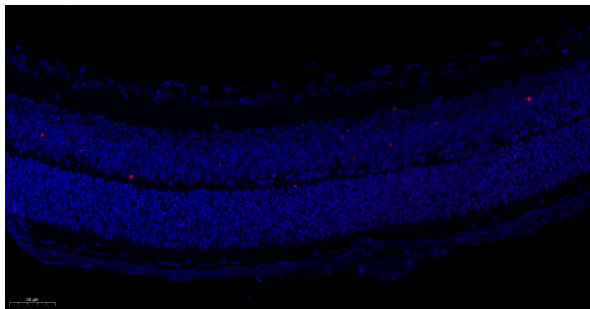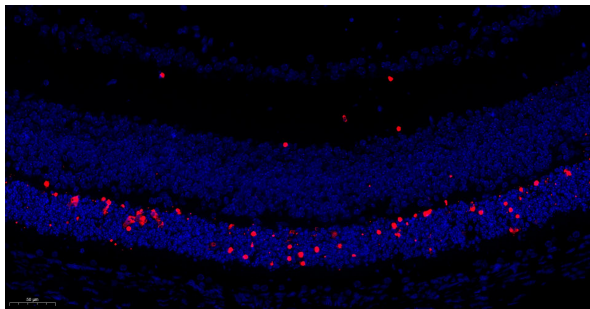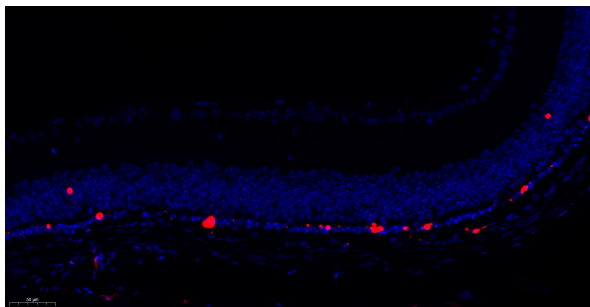

C

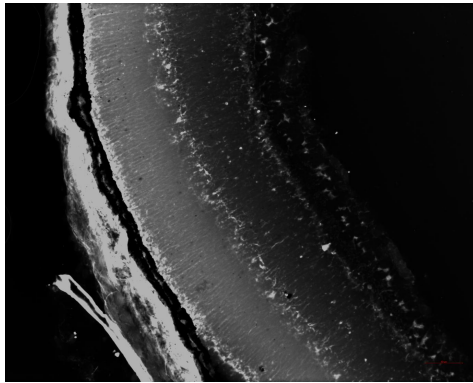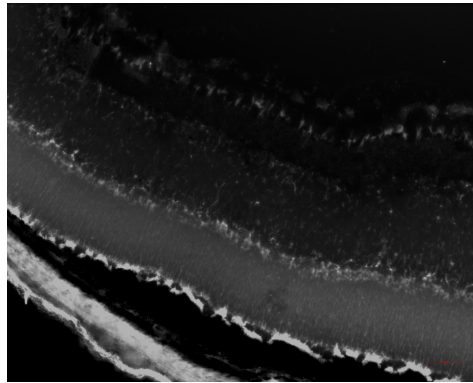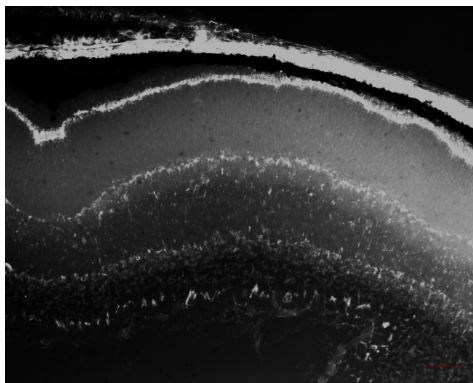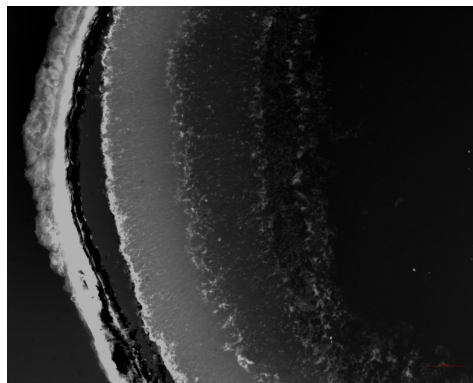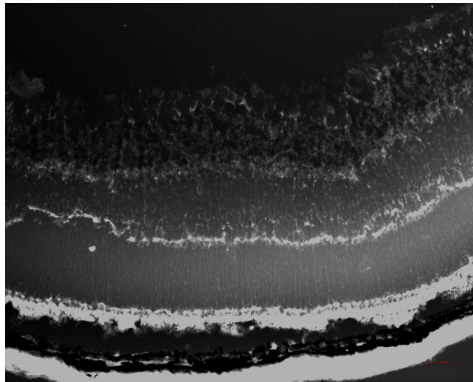

D

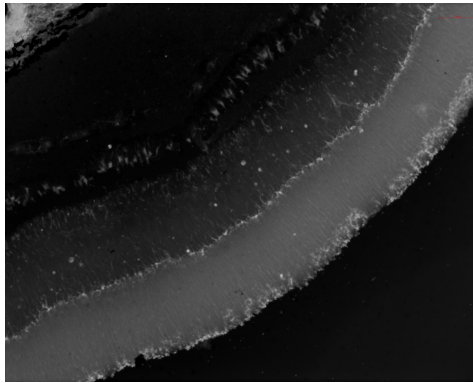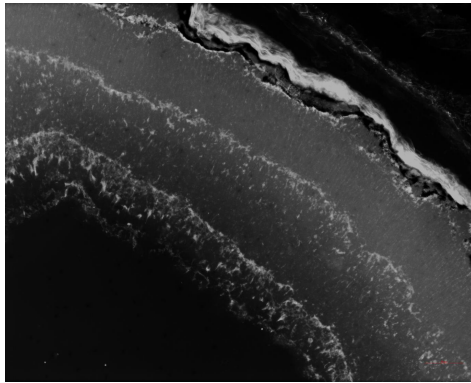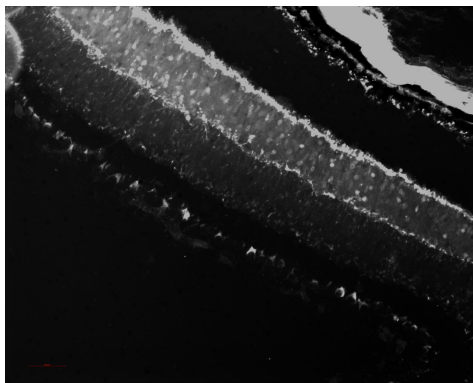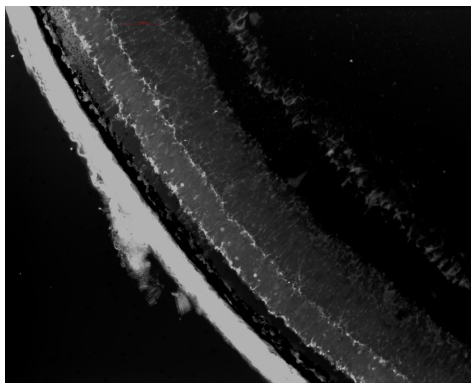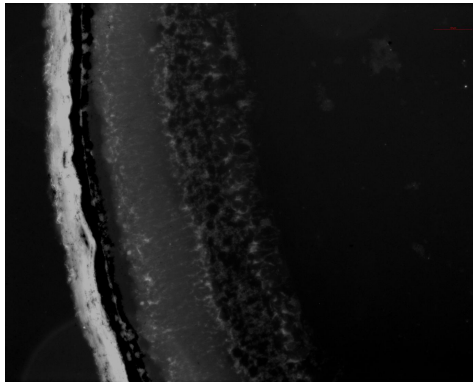

E

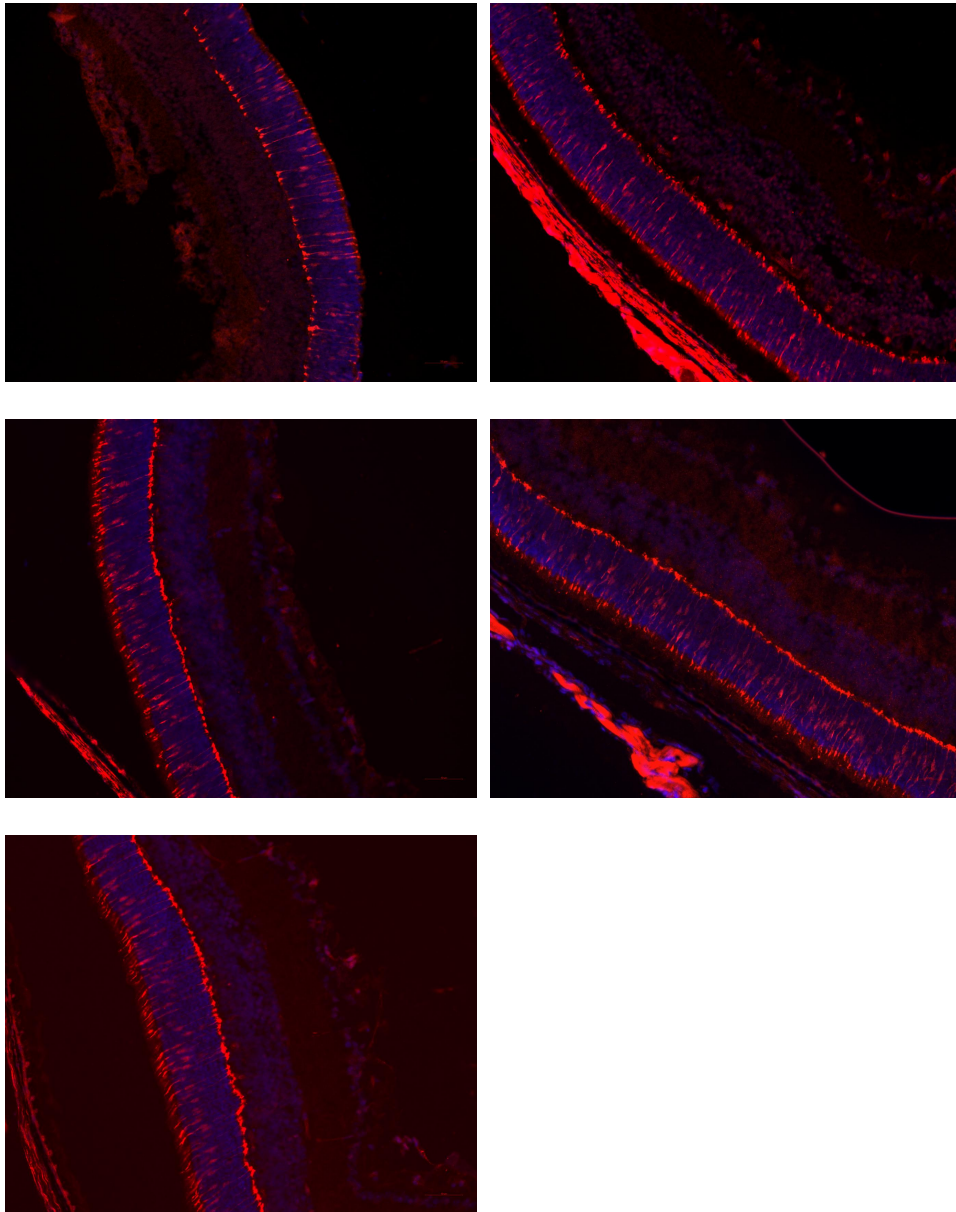

F

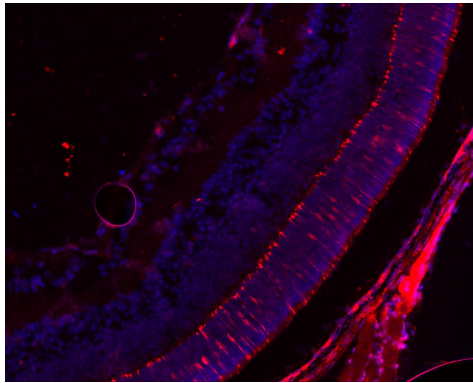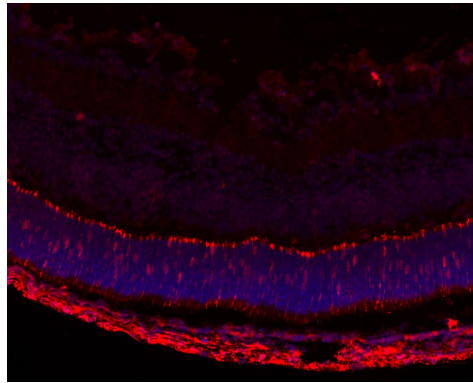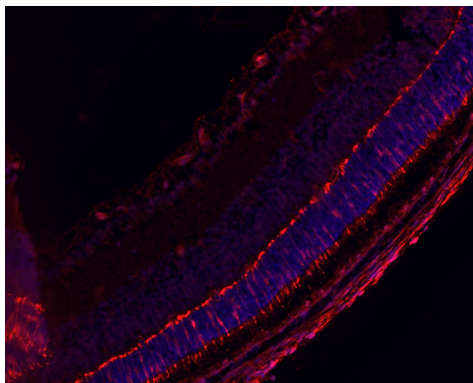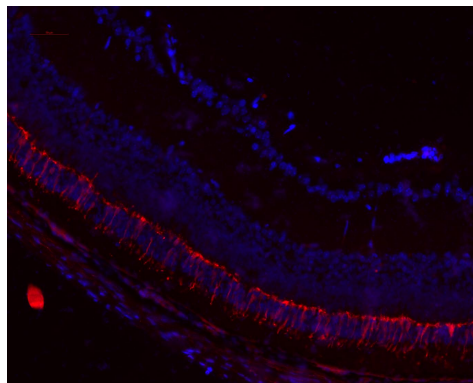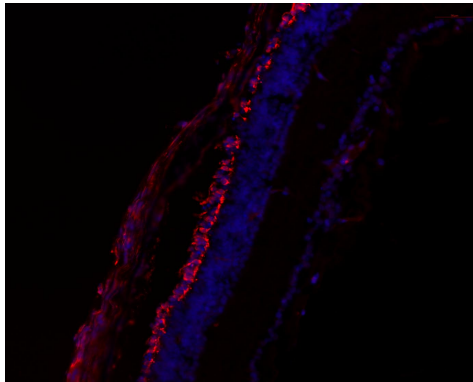

G

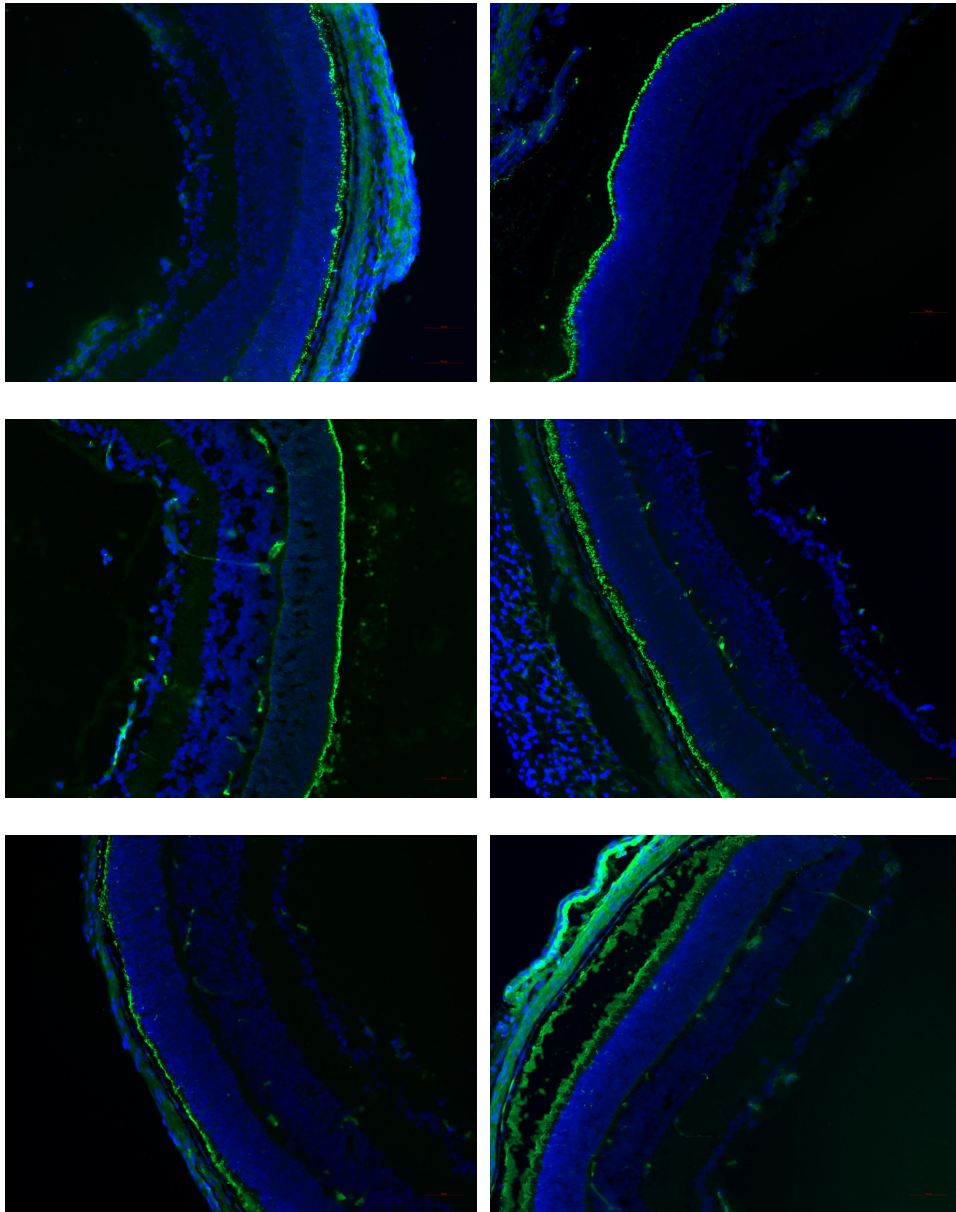

H

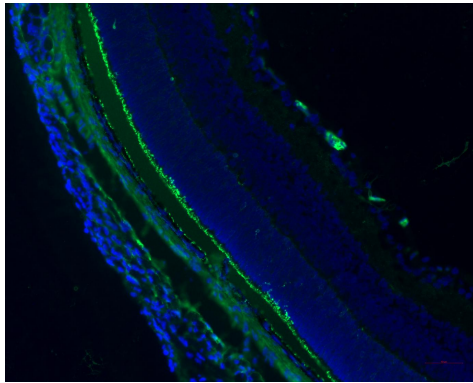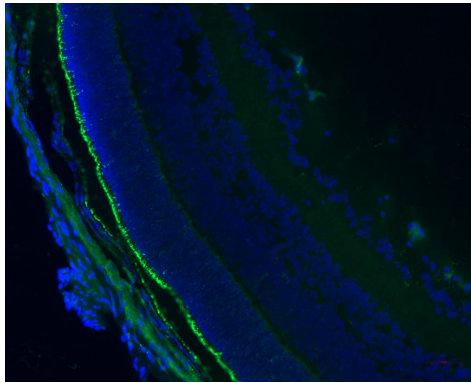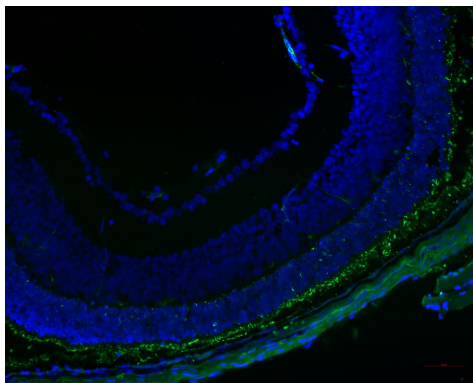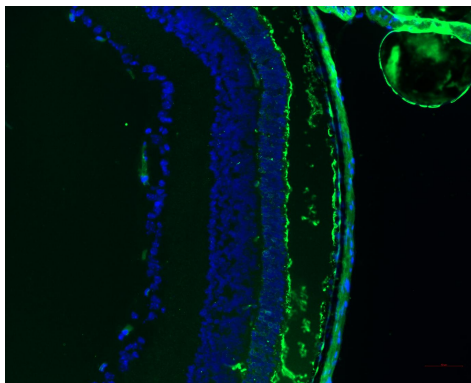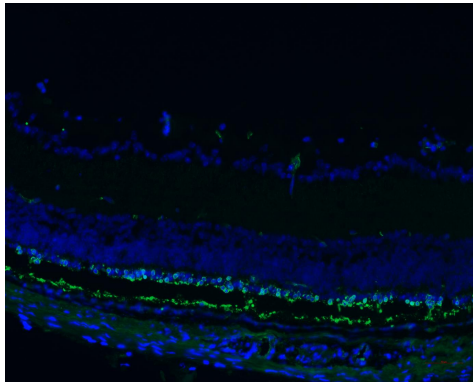

Supplement: Supplemental Information 1 [file peerj-11-15659-s001.zip › raw data/Figure 3.pdf]

A1

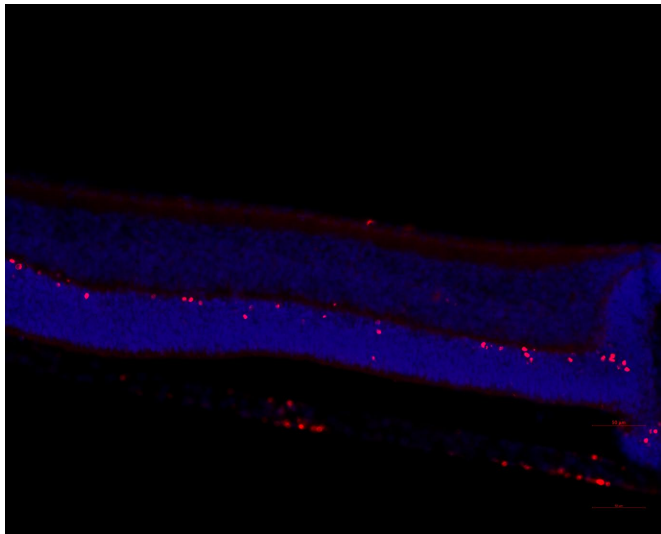

A2

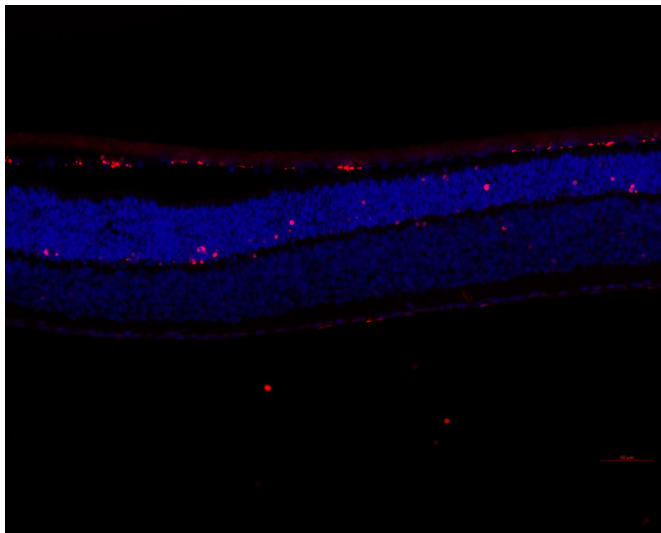

A3

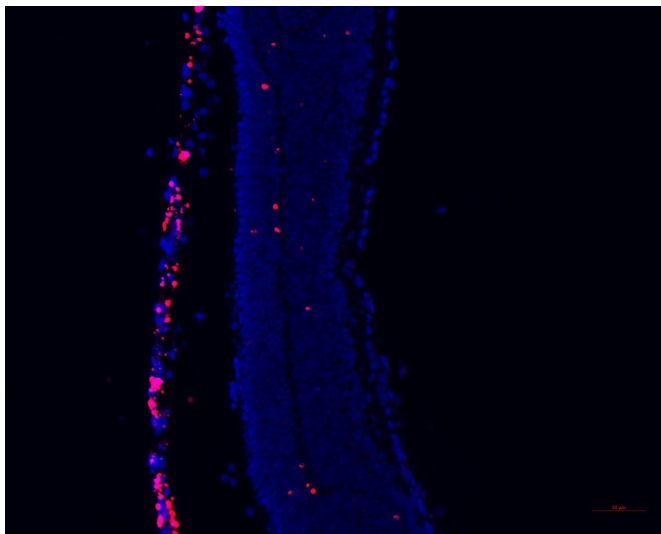

A4

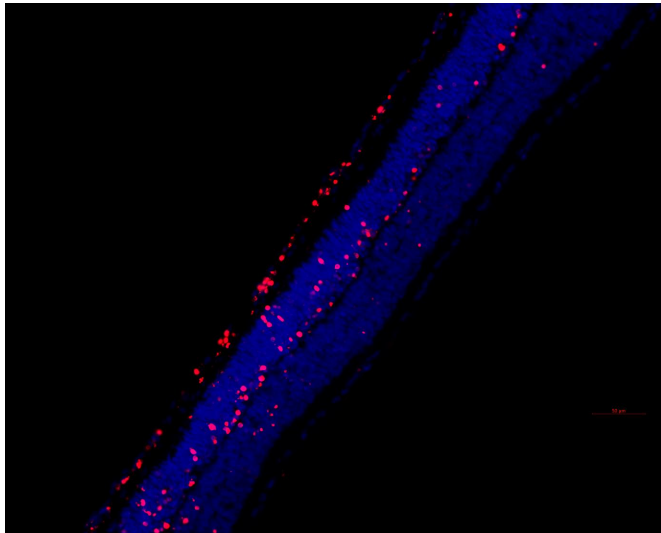

A5

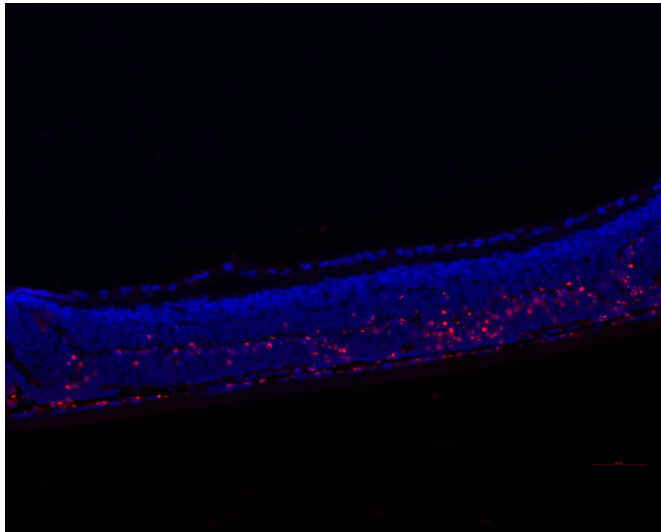

A6

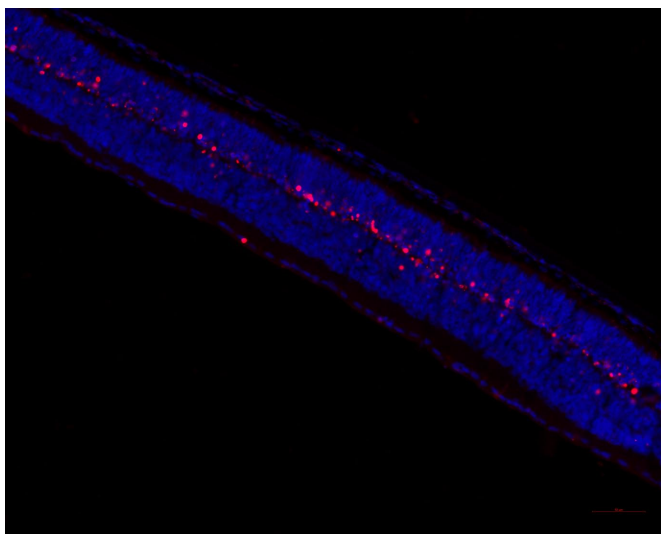

A7

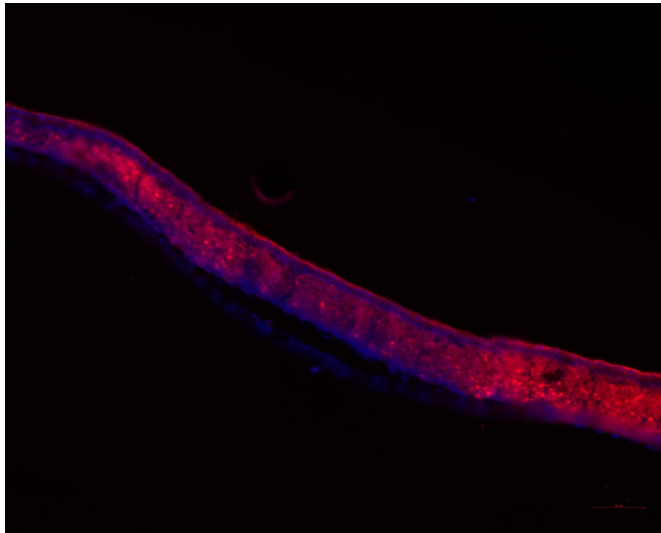

A8

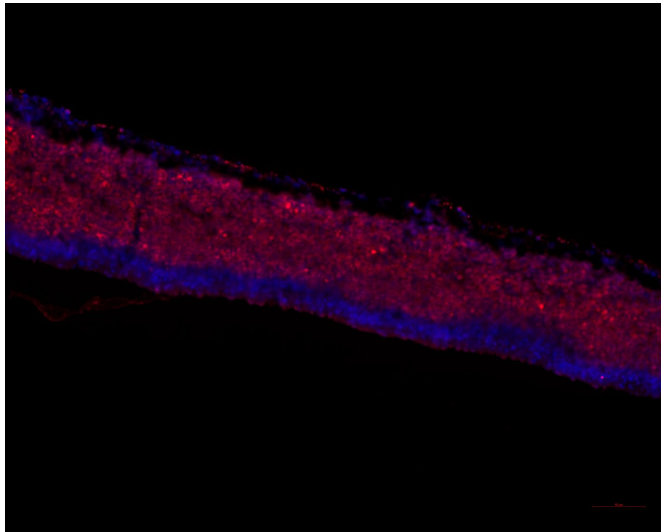

Supplement: Supplemental Information 1 [file peerj-11-15659-s001.zip › raw data/Figure 4.pdf]

A-Arrestin

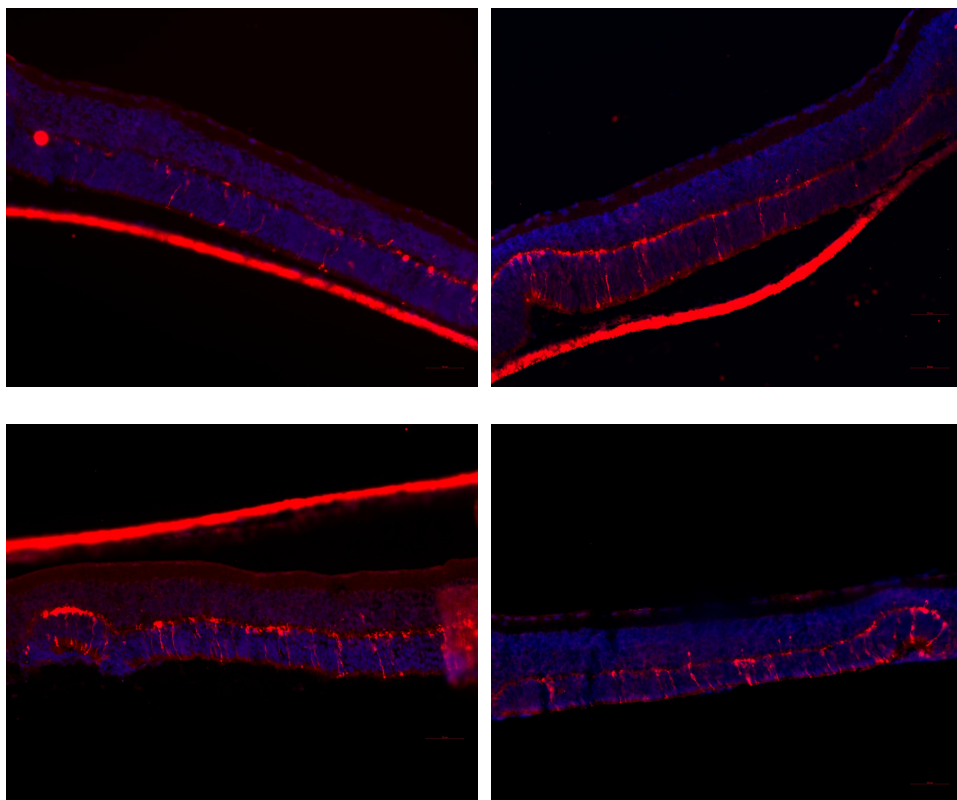

C-Rhodopsin

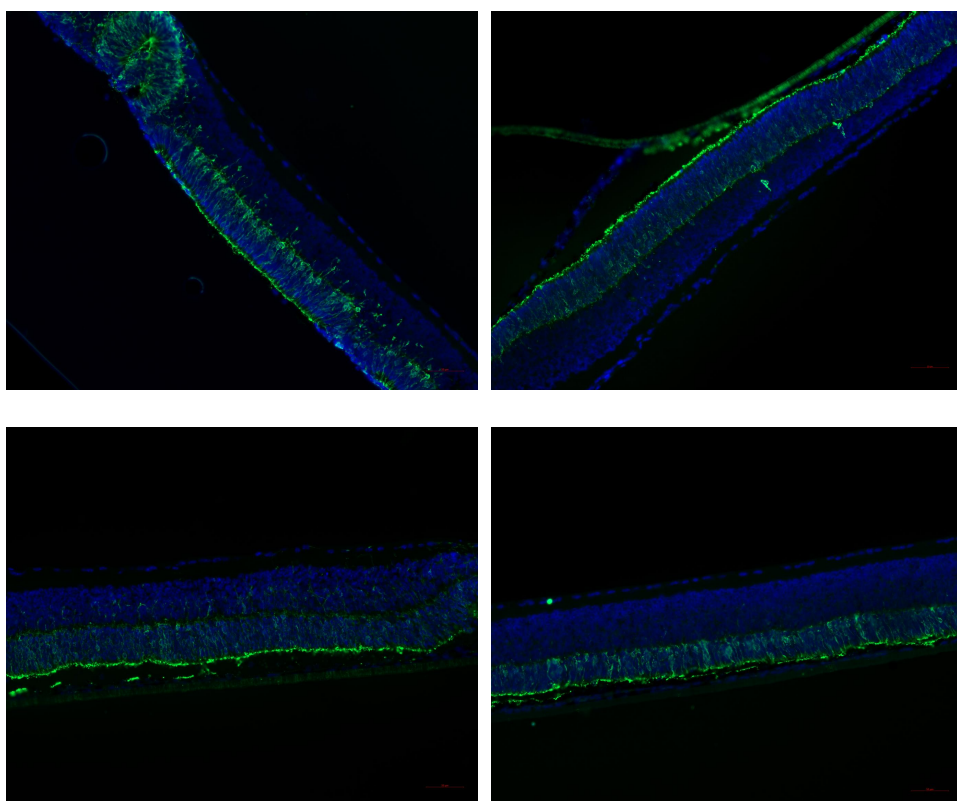

Supplement: Supplemental Information 1 [file peerj-11-15659-s001.zip › raw data/Figure 5.pdf]

A-HDAC

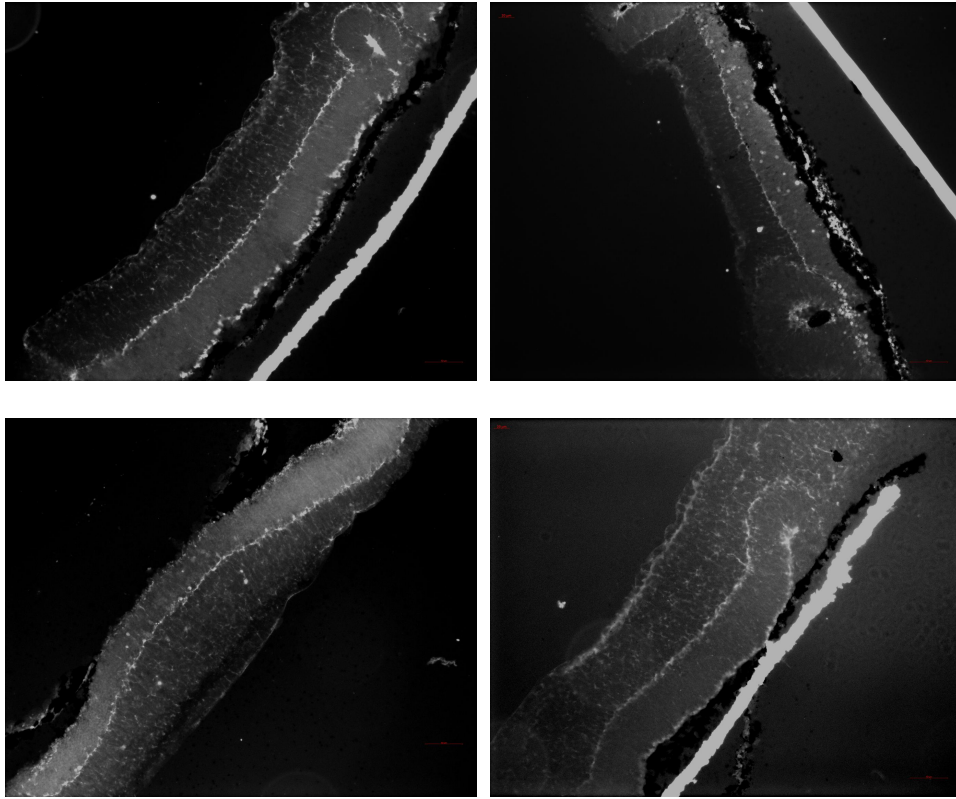

C-Parp

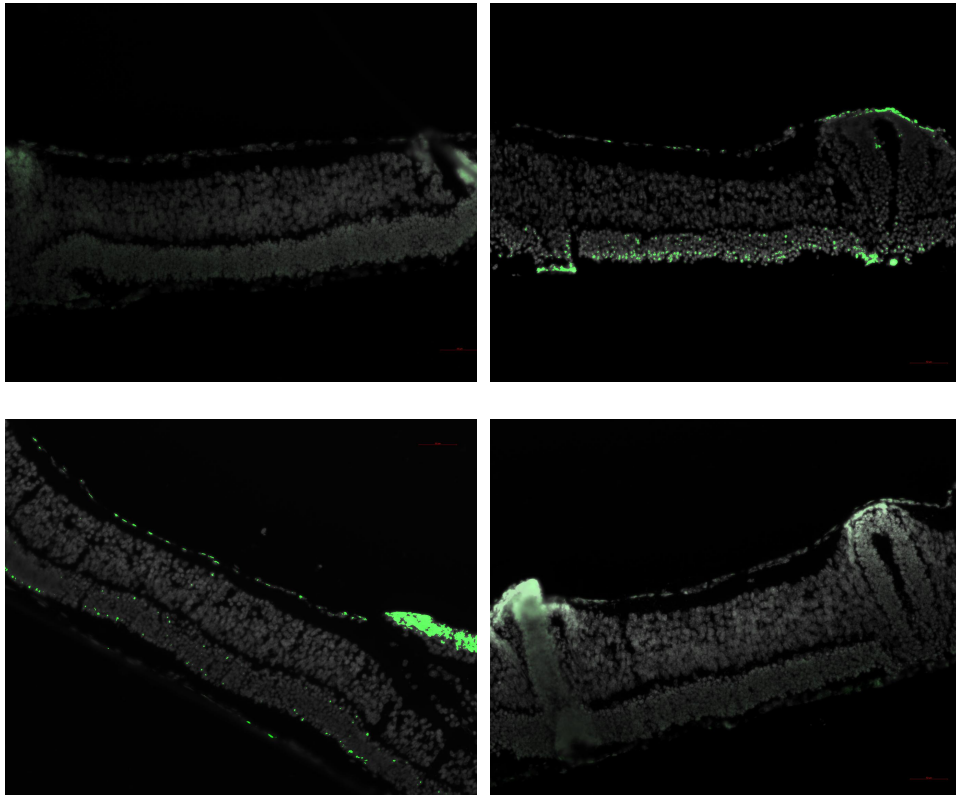

E

acH3

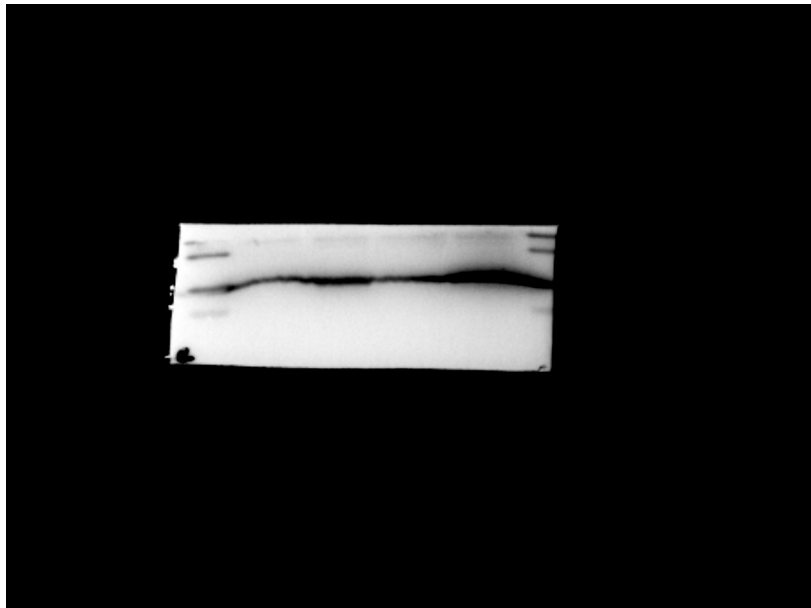

acH4

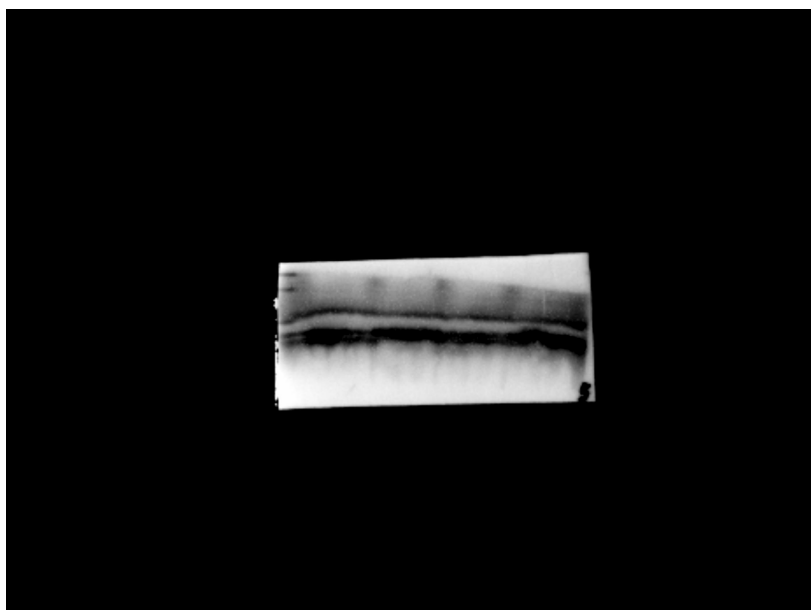

PARP1

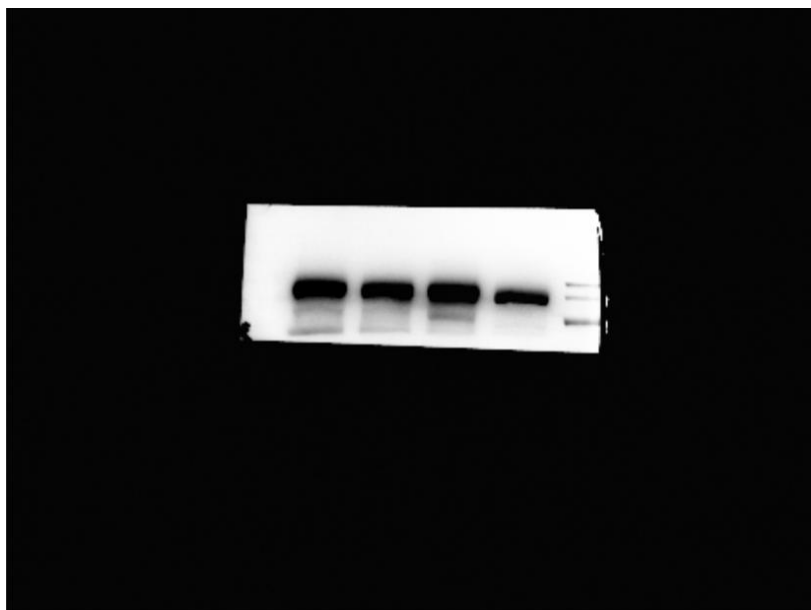

$\beta$ -actin

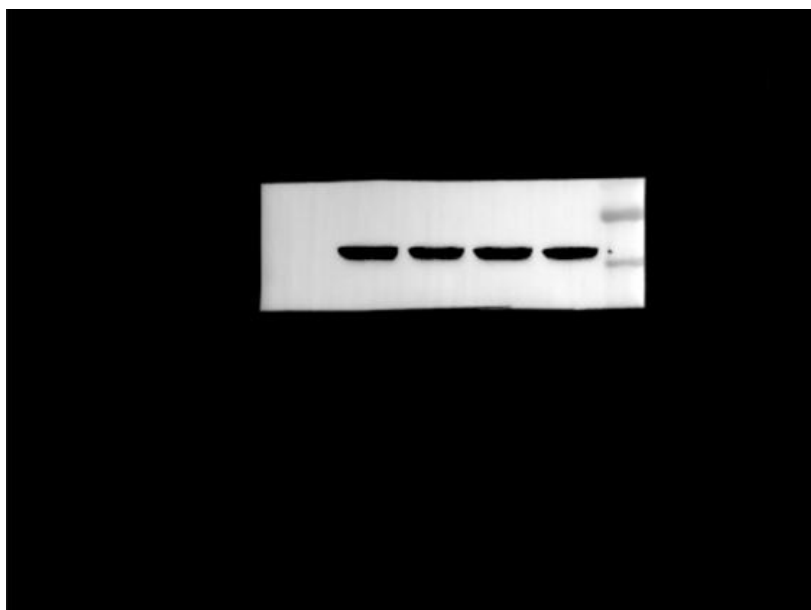

Supplement: Supplemental Information 1 [file peerj-11-15659-s001.zip › raw data/Figure 6.pdf]

acH3

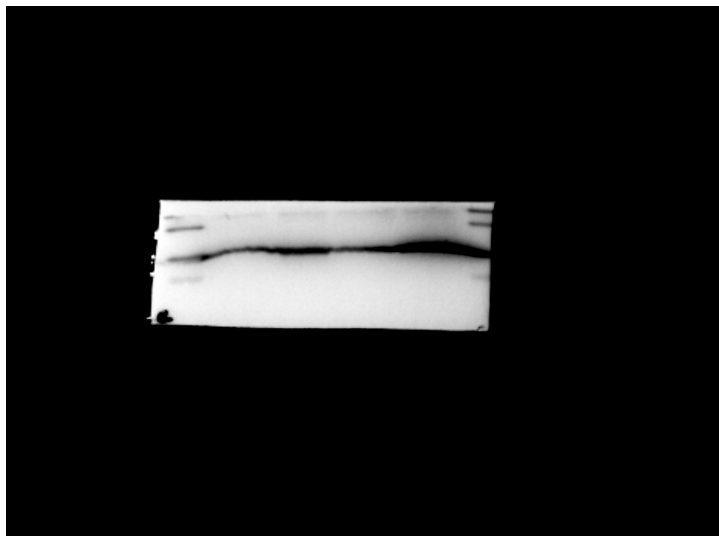

acH4

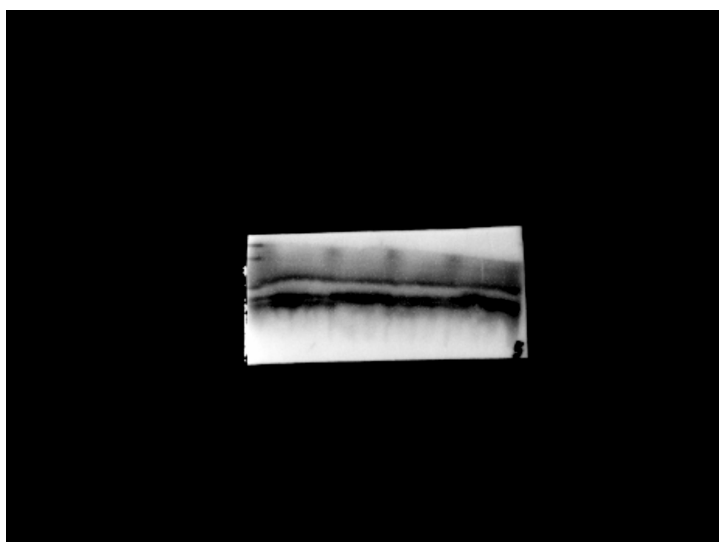

PARP1

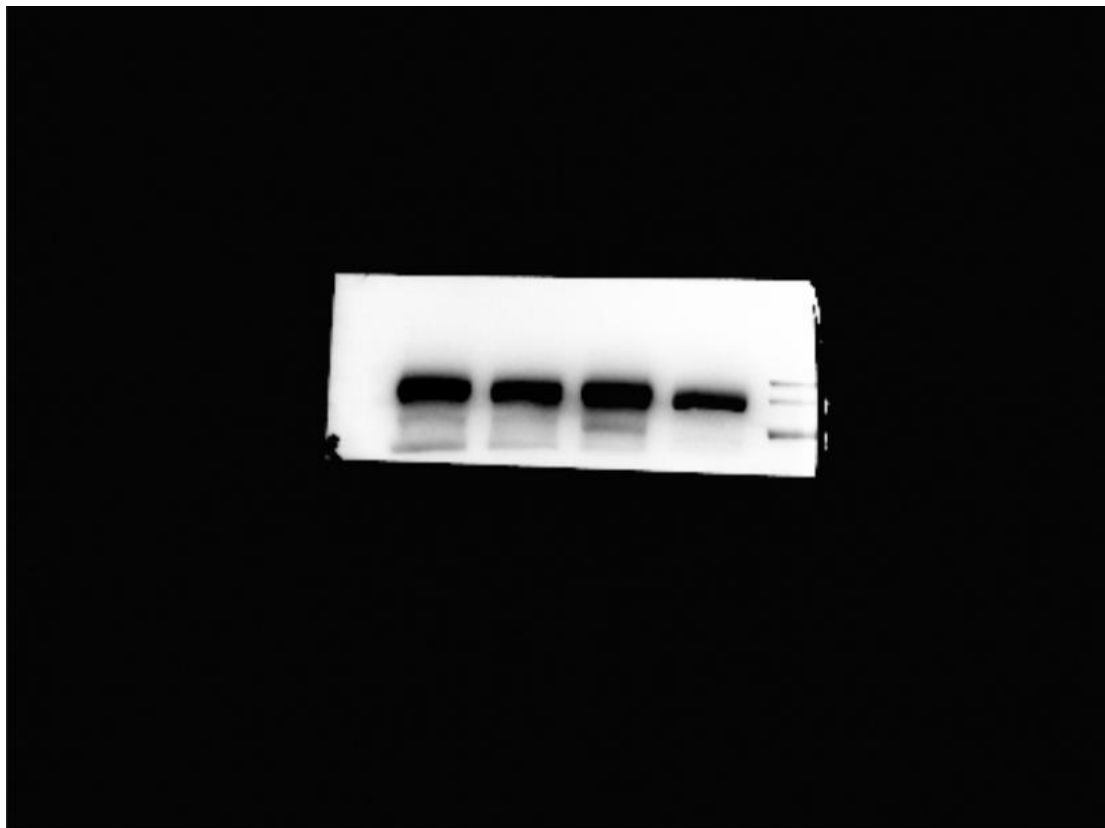

$\beta$ -actin

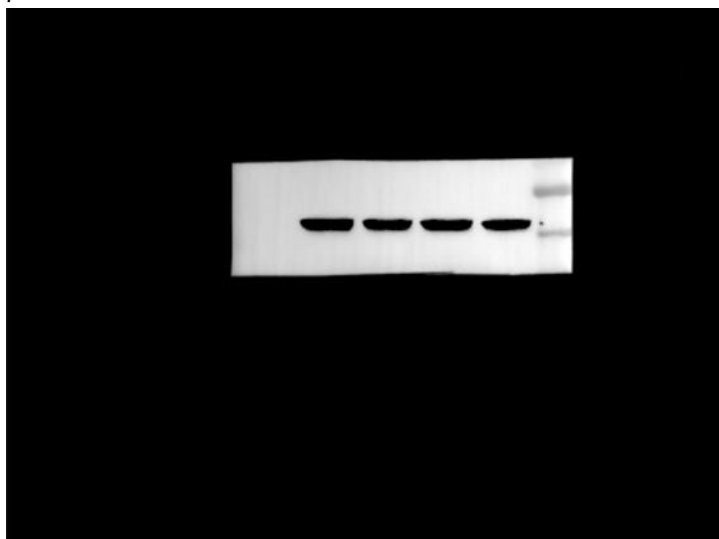

Supplement: Supplemental Information 1 [file peerj-11-15659-s001.zip › raw data/Figure 6E.pdf]

HDAC1

WT

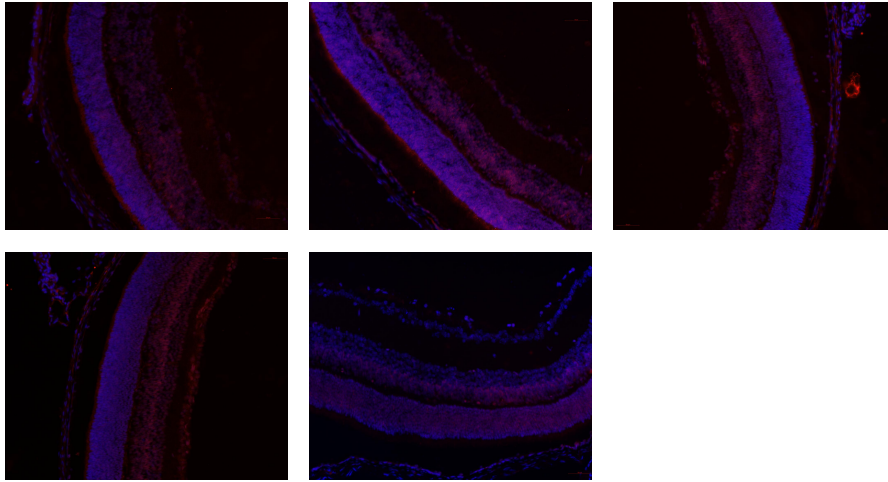

Rdl

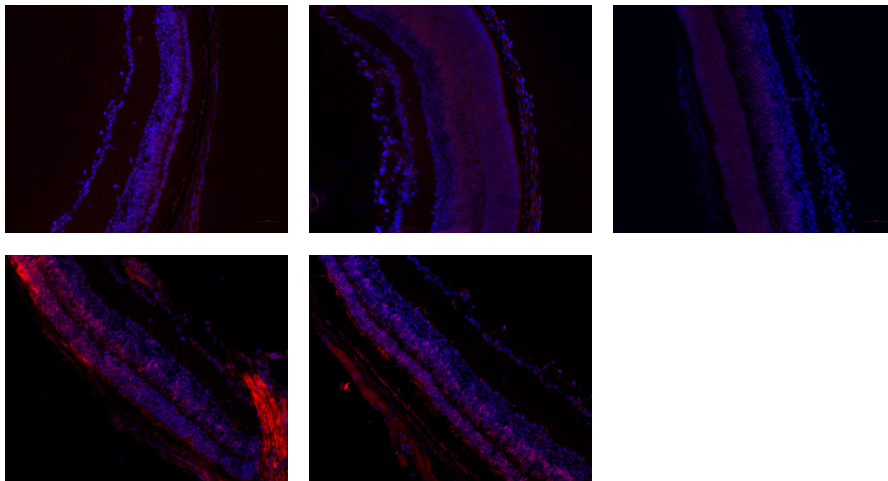

HDAC2

WT

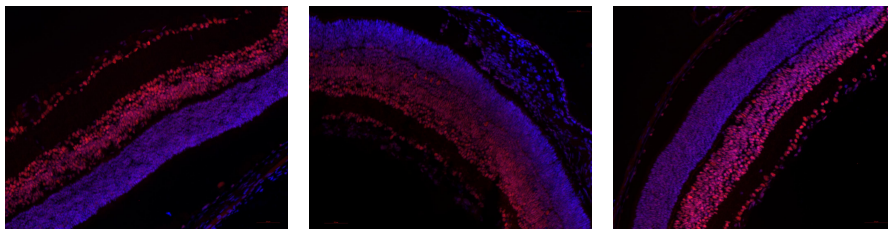

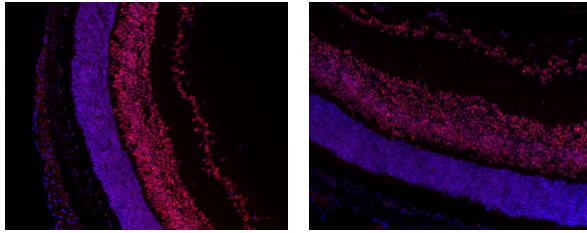

Rdl

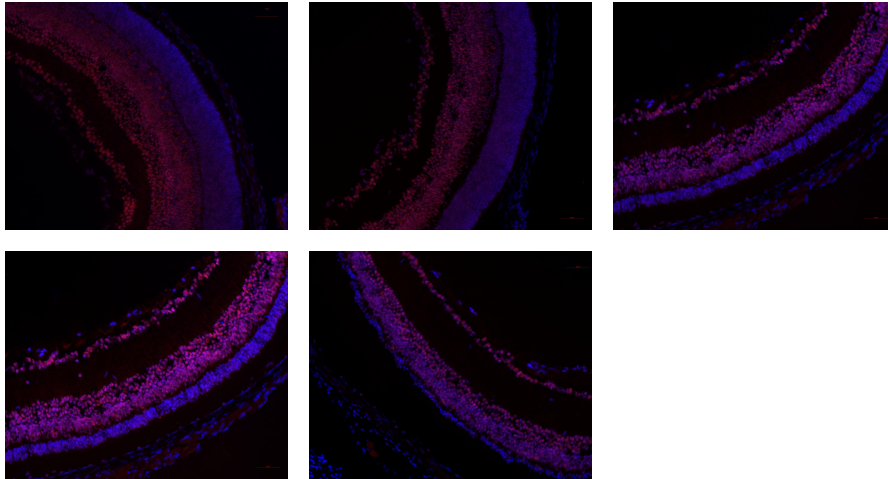

HDAC9

WT

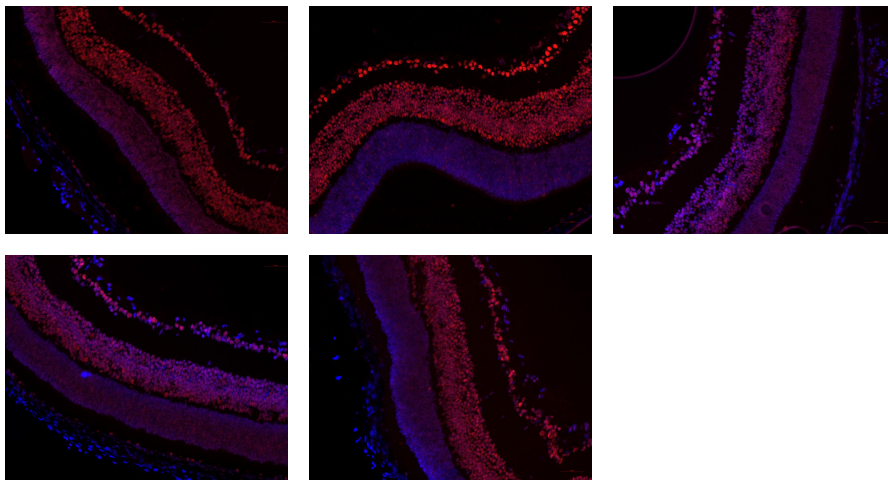

Rdl

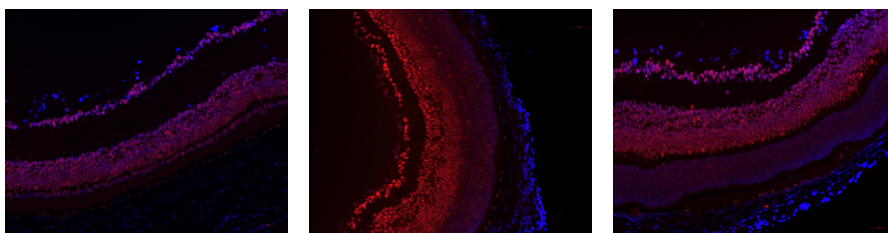

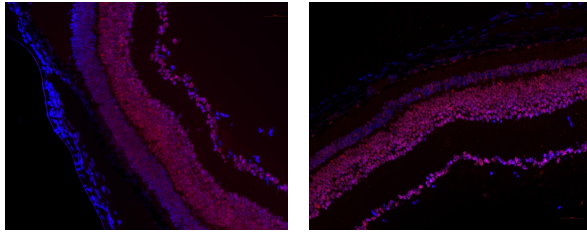

HDAC11

WT

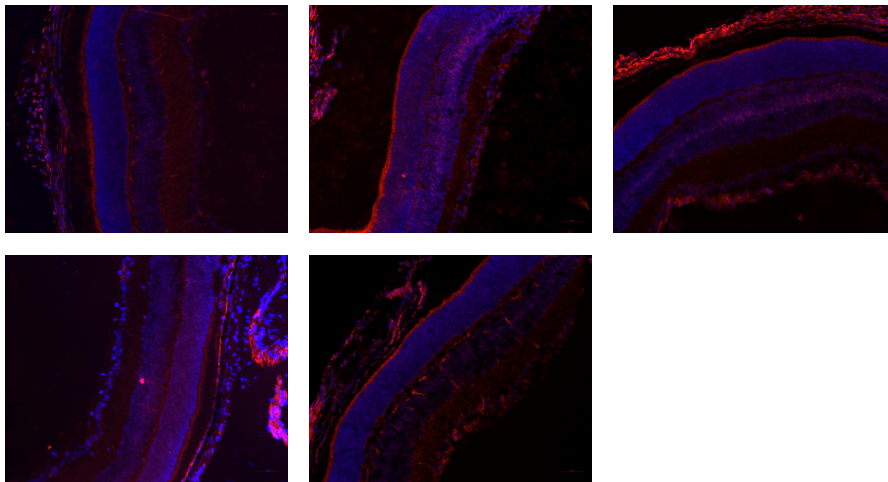

Rdl

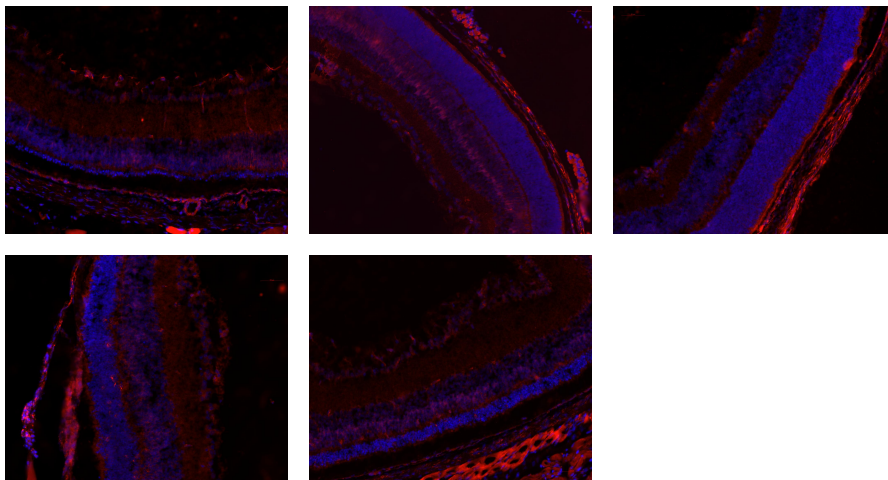

Supplement: Supplemental Information 1 [file peerj-11-15659-s001.zip › raw data/Figure S3.pdf]

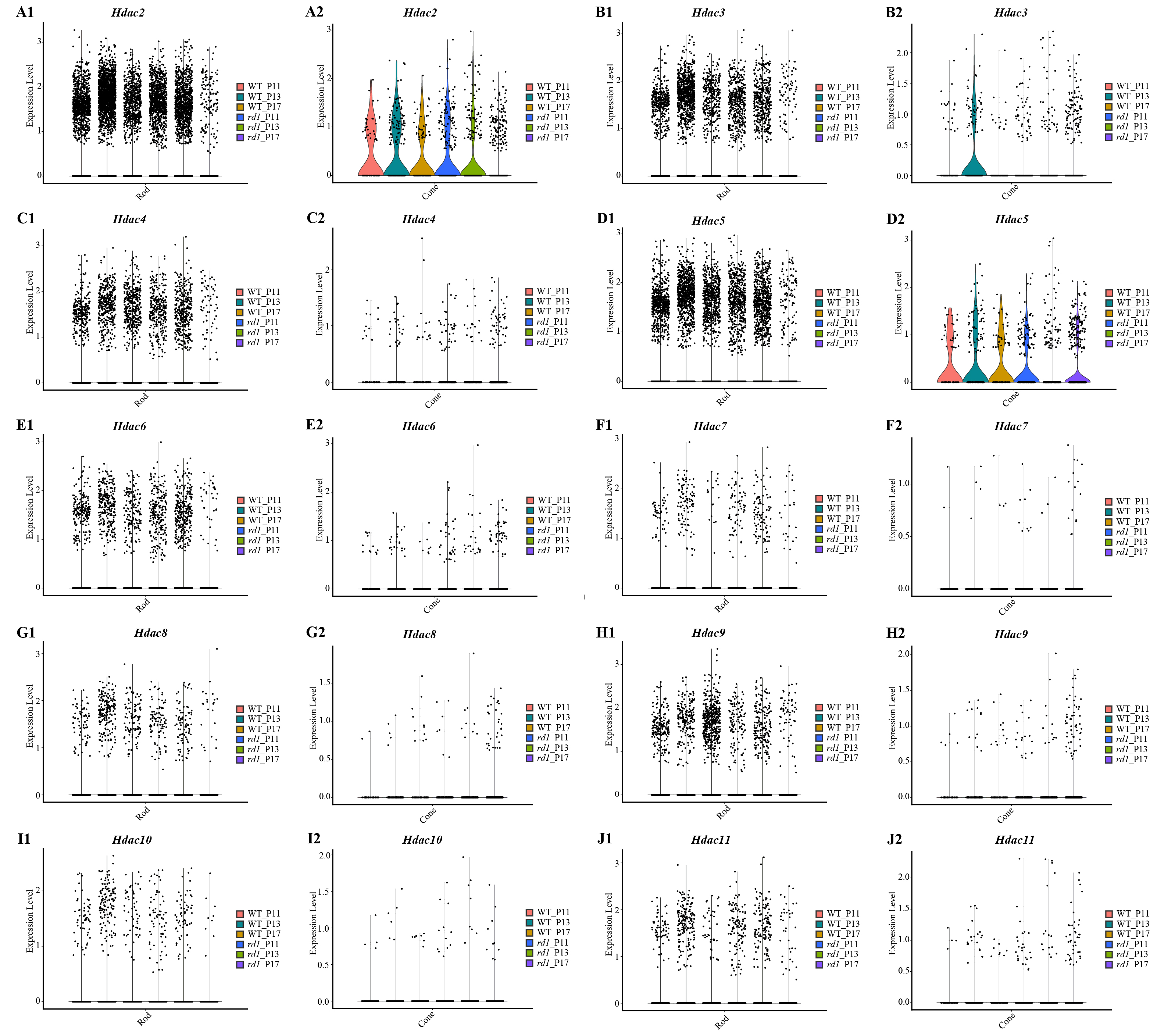

Supplement: Supplemental Information 2 — Hdac2 (A1 in rods A2 in cones), Hdac3 (B1 in rods, B2 in cones), Hdac4 (C1 in rods, C2 in cones), Hdac5 (D1 in rods, D2 in cones), Hdac6 (E1 in rods, E2 in cones), Hdac7 (F1 in rods, F2 in cones), Hdac8 (G1 in rods, G2 in cones), Hdac9 (H1 in rods, H2 in cones), Hdac10 (I1 in rods, I2 in cones), and Hdac11 (J1 in rods, J2 in cones) of different time points (P11, P13 and P17) of WT and rd1 are indicated in the violin plot. [file peerj-11-15659-s002.jpg]

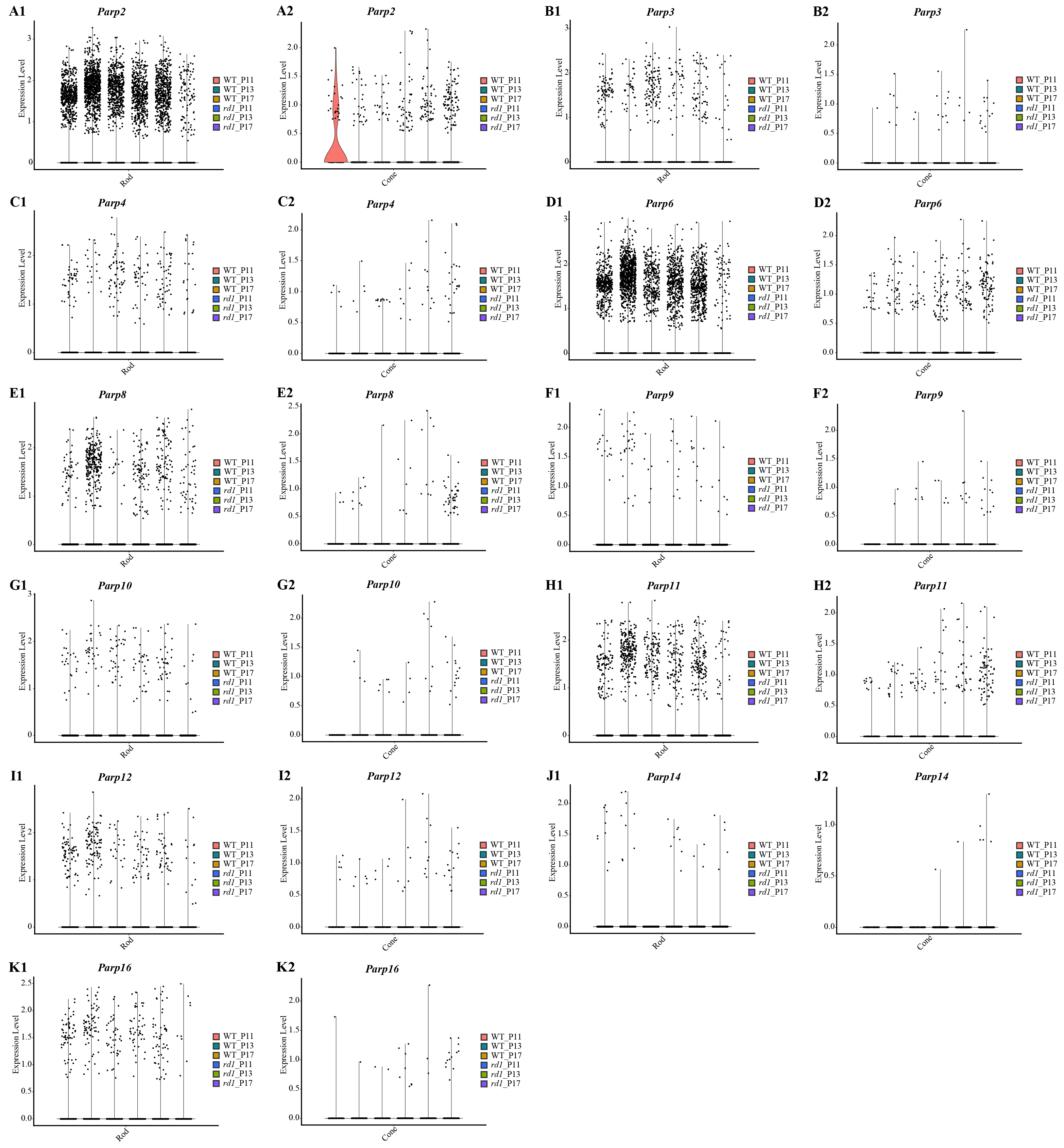

Supplement: Supplemental Information 3 — Parp2 (A1 in rods A2 in cones), Parp3 (B1 in rods, B2 in cones), Parp4 (C1 in rods, C2 in cones), Parp6 (D1 in rods, D2 in cones), Parp8 (E1 in rods, E2 in cones), Parp9 (F1 in rods, F2 in cones), Parp10 (G1 in rods, G2 in cones), Parp11 (H1 in rods, H2 in cones), Parp12 (I1 in rods, I2 in cones), Parp14 (J1 in rods, J2 in cones), and Parp16 (K1 in rods, K2 in cones) of different time points (P11, P13 and P17) of WT and rd1 are indicated in the violin plot. [file peerj-11-15659-s003.jpg]

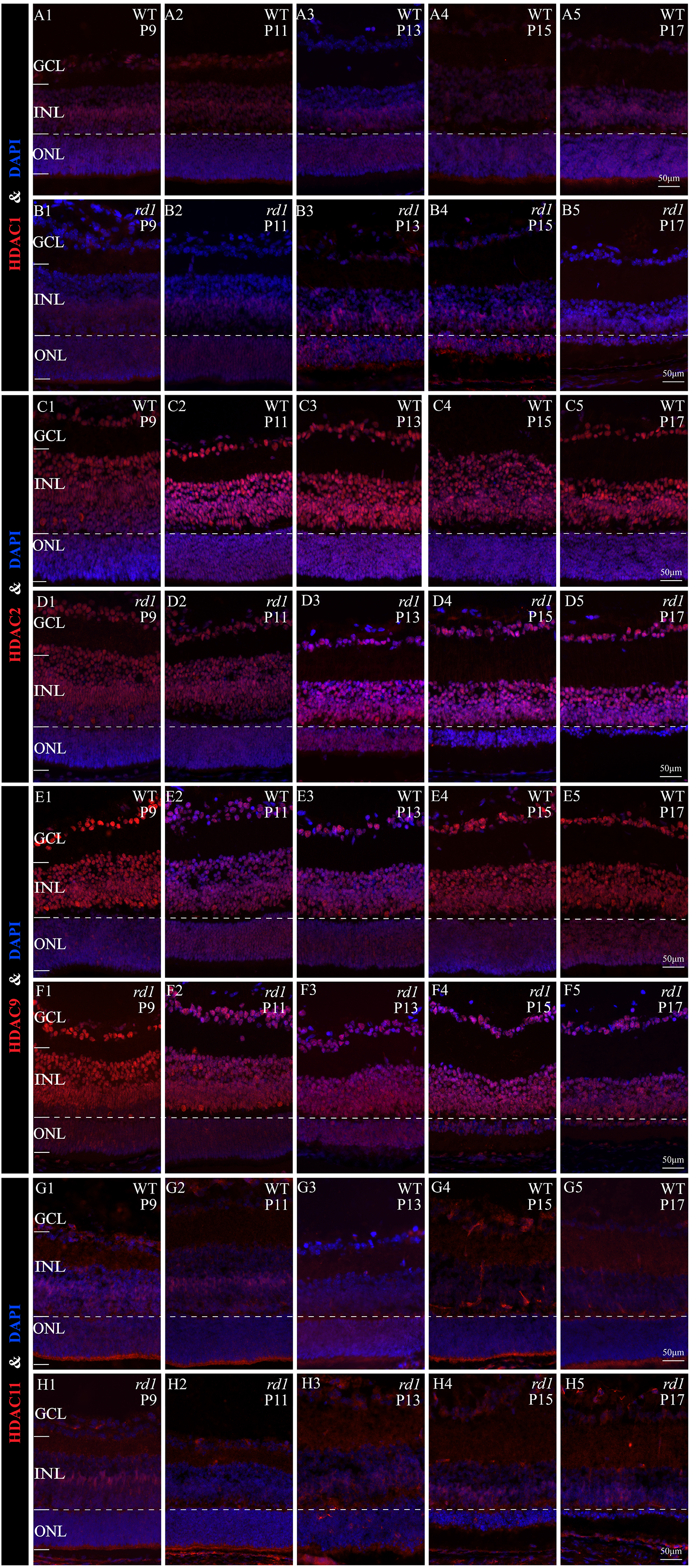

Supplement: Supplemental Information 4 — (A1-A5, B1-B5) HDAC1 positive cell (red) in WT and rd1 mice at different time points. (C1-C5, D1-D5) HDAC2 positive cell (red) in WT and rd1 mice at different time points. (E1-E5, F1-F5) HDAC9 positive cell (red) in WT and rd1 mice at different time points. (G1-G5, H1-H5) HDAC11 positive cell (red) in WT and rd1 mice at different time points.DAPI (blue) was used as a nuclear counterstain. Images shown are representative of observations for at least three different specimens of each genotype. INL, inner nuclear layer; ONL, outer nuclear layer; GCL, ganglion cell layer. Scale bar = 50 µm. [file peerj-11-15659-s004.jpg]

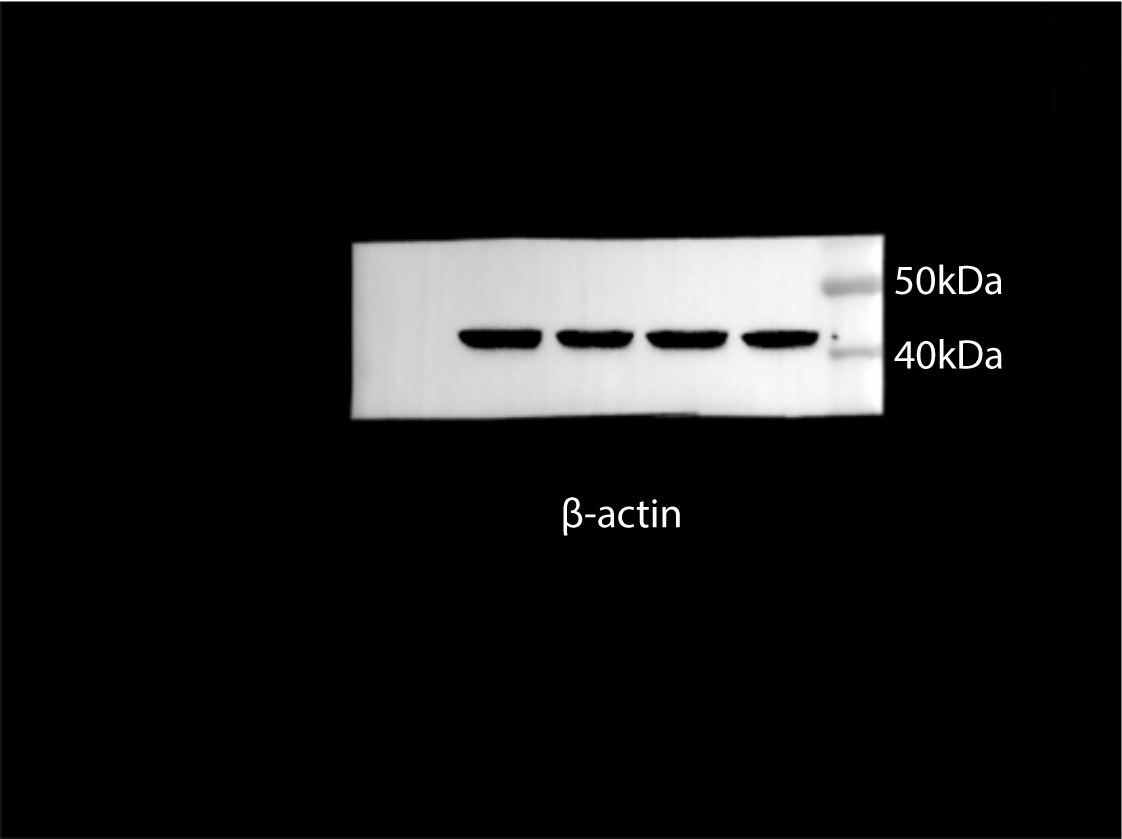

Supplement: Supplemental Information 7 [file peerj-11-15659-s007.tif]

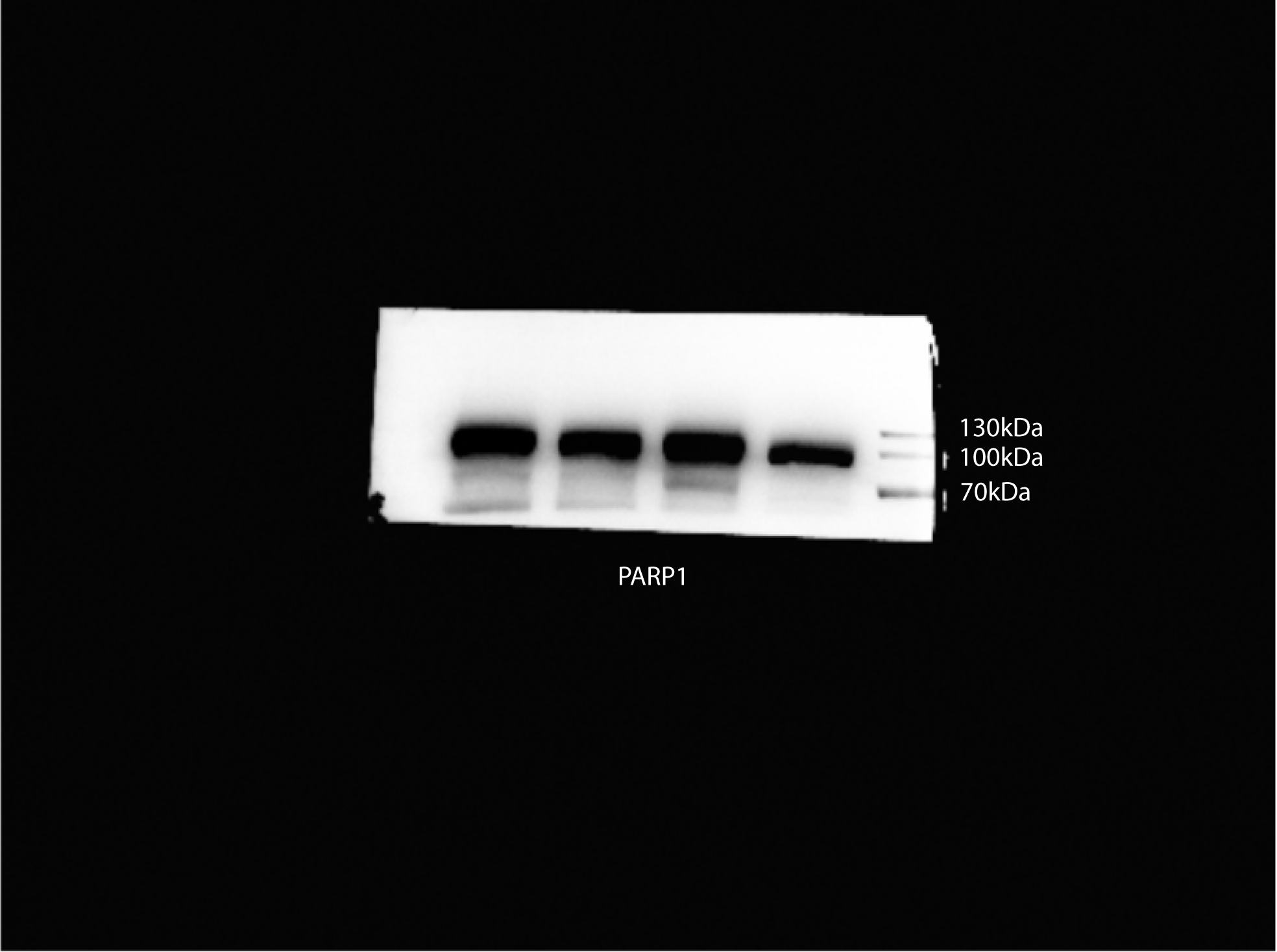

Supplement: Supplemental Information 8 [file peerj-11-15659-s008.tif]

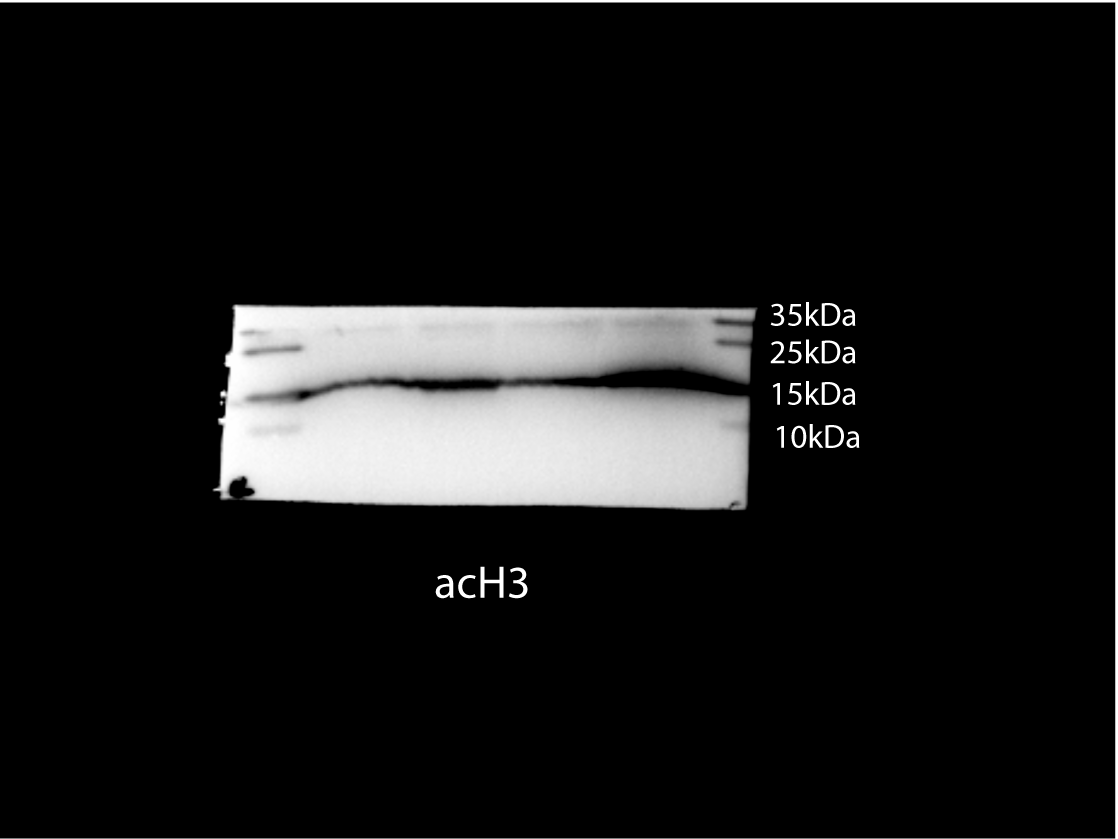

Supplement: Supplemental Information 9 [file peerj-11-15659-s009.tif]

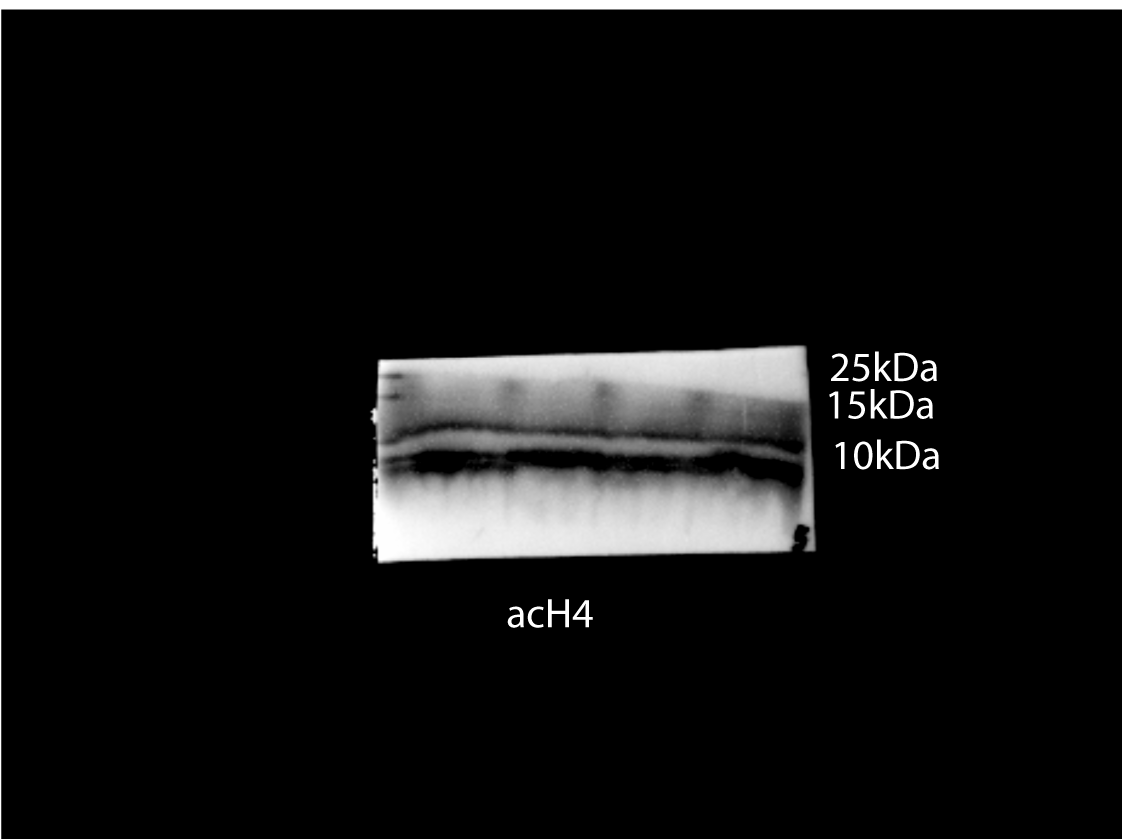

Supplement: Supplemental Information 10 [file peerj-11-15659-s010.tif]
