# Supplementary material for: HDAC inhibition delays photoreceptor loss in Pde6b mutant mice of retinitis pigmentosa: insights from scRNA-seq and CUT&Tag
Source: PeerJ. 2023 Jul 12;11:e15659. doi: 10.7717/peerj.15659 (PMC10349563; doi:10.7717/peerj.15659)
Supplement: Supplemental Information 6 [file peerj-11-15659-s006.docx]

Table S2. The overlapping 13 downregulated and 7 upregulated DEGs between RNA-seq and CUT&Tag analyses.

| **No.** | **Downregulated DEGs** | **Upregulated DEGs** |
| --- | --- | --- |
| 1 | Bend6 | 9130227L01Rik |
| 2 | C1ql2 | A930031H19Rik |
| 3 | Gfra2 | D630036H23Rik |
| 4 | Il1rapl2 | Nkx6-2 |
| 5 | Kcna1 | Rncr4 |
| 6 | Kctd16 | Taf4b |
| 7 | Pglyrp1 | Ttc30b |
| 8 | Ptprc |  |
| 9 | Rasgrf1 |  |
| 10 | Slc15a3 |  |
| 11 | Tfr2 |  |
| 12 | Tnfrsf1b |  |
| 13 | Tyrobp |  |
