# Supplementary material for: HDAC inhibition delays photoreceptor loss in Pde6b mutant mice of retinitis pigmentosa: insights from scRNA-seq and CUT&Tag
Source: PeerJ. 2023 Jul 12;11:e15659. doi: 10.7717/peerj.15659 (PMC10349563; doi:10.7717/peerj.15659)
Supplement: Supplemental Information 1 [file peerj-11-15659-s001.zip › raw data/Figure 1.doc]

Single-Cell RNA-seq (scRNA-seq) Data Availability Link: GSE212183, https://www.ncbi.nlm.nih.gov/geo/query/acc.cgi?acc=GSE212183 (accessed on 19 October 2022).
